# Prognostic and Treatment Guiding Significance of MRI-Based Tumor Burden Features and Nodal Necrosis in Nasopharyngeal Carcinoma

**DOI:** 10.3389/fonc.2020.537318

**Published:** 2020-09-11

**Authors:** Xi Chen, Xun Cao, Bingzhong Jing, Weixiong Xia, Liangru Ke, Yanqun Xiang, Kuiyuan Liu, Mengyun Qiang, Chixiong Liang, Jianpeng Li, Mingyong Gao, Wangzhong Li, Jingjing Miao, Guoying Liu, Zhuochen Cai, Shuhui Lv, Xiang Guo, Chaofeng Li, Xing Lv

**Affiliations:** ^1^State Key Laboratory of Oncology in South China, Collaborative Innovation Center for Cancer Medicine, Department of Nasopharyngeal Carcinoma, Sun Yat-sen University Cancer Center, Guangzhou, China; ^2^State Key Laboratory of Oncology in South China, Collaborative Innovation Center for Cancer Medicine, Intensive Care Unit, Sun Yat-sen University Cancer Center, Guangzhou, China; ^3^State Key Laboratory of Oncology in South China, Collaborative Innovation Center for Cancer Medicine, Department of Information Technology, Sun Yat-sen University Cancer Center, Guangzhou, China; ^4^State Key Laboratory of Oncology in South China, Collaborative Innovation Center for Cancer Medicine, Department of Medical Imaging, Sun Yat-sen University Cancer Center, Guangzhou, China; ^5^Department of Radiology, Dongguan People’s Hospital, Dongguan, China; ^6^Department of Medical Imaging, The First People’s Hospital of Foshan, Foshan, China

**Keywords:** MRI-based tumor burden features, nodal necrosis, distant metastasis, treatment, nasopharyngeal carcinoma

## Abstract

We aimed to develop a nomogram integrating MRI-based tumor burden features (MTBF), nodal necrosis, and some clinical factors to forecast the distant metastasis-free survival (DMFS) of patients suffering from non-metastatic nasopharyngeal carcinoma (NPC). A total of 1640 patients treated at Sun Yat-sen University Cancer Center (Guangzhou, China) from 2011 to 2016 were enrolled, among which 1148 and 492 patients were randomized to a training cohort and an internal validation cohort, respectively. Additionally, 200 and 257 patients were enrolled in the Foshan and Dongguan validation cohorts, respectively, which served as independent external validation cohorts. The MTBF were developed from the stepwise regression of six multidimensional tumor burden variables, based on which we developed a nomogram also integrating nodal necrosis and clinical features. This model divided the patients into high- and low-risk groups by an optimal cutoff. Compared with those of patients in the low-risk group, the DMFS [hazard ratio (HR): 4.76, 95% confidence interval (CI): 3.39–6.69; *p* < 0.0001], and progression-free survival (PFS; HR: 4.11, 95% CI: 3.13–5.39; *p* < 0.0001) of patients in the high-risk group were relatively poor. Furthermore, in the training cohort, the 3-year DMFS of high-risk patients who received induction chemotherapy (ICT) combined with concurrent chemoradiotherapy (CCRT) was better than that of those who were treated with CCRT alone (*p* = 0.0340), whereas low-risk patients who received ICT + CCRT had a similar DMFS to those who only received CCRT. The outcomes we obtained were all verified in the three validation cohorts. The survival model can be used as a reliable prognostic tool for NPC patients and is helpful to determine patients who will benefit from ICT.

## Introduction

Nasopharyngeal carcinoma (NPC) is a typical disease, of which the incidence rate is the highest in Southeast Asia (1). In the early stage, the tumor can be successfully controlled by radiotherapy alone (2), and locoregionally advanced NPC is recommended to be treated with concurrent chemotherapy (3, 4). It has been reported that the 5-year overall survival (OS) has reached 85% (4, 5). However, NPC is prone to recurrence and/or metastasis after certain treatments, which is the main cause of treatment failure and the major cause of mortality of NPC patients (6–8).

In addition, patients in the same clinical stage receiving similar treatments have different survival outcomes. Therefore, it is necessary to build an effective prognostic model to identify patients with a poor prognosis for whom intensive follow-up and adjuvant chemotherapy may bring about more survival benefits. Thus, many efforts have been made to identify risk factors ranging from biomarkers, such as Epstein–Barr virus (EBV) DNA and gene expression, to radiomics (9–13). Multiplanar magnetic resonance imaging (MRI) is one of the methods most extensively used for the precise mapping of the tumor and the accurate evaluation of NPC. In addition to precisely mapping the tumor and defining the T and N stages of NPC, attempts have been made to include information from MRI into the prognostic analysis of cancer (12, 14–17).

Although tumor volume and nodal volume are commonly acknowledged prognostic tumor burden factors (18–20), NPC is an irregular tumor that shows an expansive, infiltrating, or mixed growth pattern. For the expansive growth pattern, tumor volume is highly associated with T stage, while for the infiltrating growth pattern, it is less associated. The nasopharynx is in the subjacent skull base and has a complex anatomy; invasive NPC can infiltrate the cranial nerves through structural channels such as the pterygopalatine fossa, inferior orbital fissure, ethmoid sinus, and foramen lacerum (21–23). NPC patients with small tumor volumes but large extents of invasion might have poor prognosis. In addition to tumor and nodal volume, more information is needed to precisely reflect the tumor burden. Certain prognostic-related structural information reflecting tumor burden, such as the sectional area and vertical dimension, is not being fully utilized. At the same time, induction chemotherapy (ICT) has been shown to be efficient with low toxicity (24–26); however, not all patients benefit from ICT. A more effective prediction model is needed to identify low-risk patients to reduce overtreatment when concurrent chemoradiotherapy (CCRT) would be enough. As previous studies have revealed that nodal necrosis and nodal level are important imaging features and are independent negative prognostic factors for NPC (14, 27–29), we also collected nodal necrosis and nodal level data from MRI. Therefore, in this study, we established an MRI-based tumor burden feature (MTBF) model and developed a nomogram based on MTBF combined with nodal necrosis and some clinical factors to predict distant metastasis in NPC patients. Furthermore, we used this survival model to further explore the relationship between patients with a high risk of poor outcomes and the corresponding therapeutic schedule, which may help in making clinical decisions for individual patients suffering from NPC.

## Materials and Methods

### Study Design and Participants

In this retrospective study, 1640 patients with non-metastatic NPC who were treated at Sun Yat-sen University Cancer Center (Guangzhou, China) from 2011 to 2016 were enrolled. For the inclusion and exclusion criteria of the participants, see Supplementary Material p 1. A total of 1148 and 492 patients were randomized to a training cohort and an internal validation cohort, respectively, by computer-generated random numbers [random numbers from 1 to 1640 for each patient, utilizing the function “sample” in R project (version 3.5.2) with a pre-defined seed “250,” and the ratio of training cohort to internal validation cohort size is 7 to 3]. To further validate the generalizability of the model, we enrolled 200 and 257 patients into two external validation cohorts, the Foshan and Dongguan validation cohorts, respectively. The patients in these two external validation cohorts were enrolled following the same criteria. The tumor stage of the enrolled patients was determined in accordance with the 8th edition of the American Joint Committee on Cancer staging manual. This study was carried out with approval of the ethical committees of the Chinese Clinical Trial Registry (ChiECRCT20190127). We have uploaded all crucial research data to the Research Data Deposit public platform (RDDA2020001382).

### Procedures

We utilized a Microsoft Excel form to collect sociodemographic information (including age, sex, native place, weight, BMI, and height) and baseline clinical data [EBV DNA before treatment, EA-IgA, VCA-IgA, LYMPH, WBC, RBC, PLT, HGB, total protein (TP), ALB, lactate dehydrogenase (LDH), C-reactive protein (CRP), ABO blood type, RH blood type, T stage, N stage, clinical stage, and therapeutic regimen]. All variables above were categorized based on routine cutoff points in clinical findings and applications. Nodal necrosis (positive vs negative) and nodal level (above the caudal edge of cricoid cartilage vs lower) were also recorded as categorical variables. Tumor burden variables (volume, maximum cross-sectional area, and vertical dimension of the primary tumor and regional lymph nodes, of which the abbreviations are Tv and Lv, Ta and La, and Td and Ld, respectively) were also recorded in the Excel form as continuous variables.

Unenhanced and enhanced head and neck MRI of NPC participants were assessed, and the execution details are presented in Supplementary Material p 1. Based on enhanced T1-weighted imaging (T1WI), three specialists in MRI contoured the margin of the primary tumor and regional lymph nodes by utilizing Medical Imaging Interaction Toolkit (MITK) software (version MITK-2016.11.0; Supplementary Figure 1). Any disagreements were resolved by a consensus. Detailed information on the calculation of Tv, Lv, Ta, La, Td, and Ld is presented in Supplementary Materials p 1–2

### Treatment Methods

All patients underwent radiotherapy. Patients from our center and those in the Foshan validation cohort received intensity-modulated radiation therapy (IMRT). A total of 2.3% (6 of 257) of the patients in the Dongguan validation cohort received two-dimensional radiotherapy (2D-RT), and 97.7% (251 of 257) of the patients received IMRT. The cumulative radiation doses were 66 Gy or greater in 30–35 fractions, and treatment was delivered once daily, over 5 fractions per week. All patients received platinum-based chemotherapy, including concurrent chemotherapy and ICT. ICT consisted of cisplatin with 5-fluorouracil, taxanes, or both triweekly for two or three cycles. Concurrent chemotherapy consisting of cisplatin was administered weekly or triweekly during radiotherapy.

### Follow-Up and Study Endpoints

Follow-up surveys were conducted every 3 months over the first 2 years after radical therapy, once semiannually in the third to fifth years, and once yearly thereafter. The follow-up involved the determination of plasma EBV DNA concentration and indirect or direct nasopharyngoscopy, X-ray/plain and contrast-enhanced CT imaging of the chest, sonography/plain and enhanced CT of the abdomen, and plain and enhanced MRI of the head and neck. In this study, the primary endpoint was distant metastasis-free survival (DMFS), defined as the period from the first day of treatment (radiotherapy or chemotherapy) to the first occurrence of distant metastasis. The second endpoint was progression-free survival (PFS), defined as the period of time from the first diagnosis to locoregional failure, distant failure, or death from any cause, whichever occurred first.

### Statistical Analysis

We first carried out univariate analysis on the six tumor burden variables (Tv, Lv, Ta, La, Td, and Ld), in which the *p* value adopted for excluding variables with least significance was 0.1 (*p* > 0.1). Then, we conducted multivariate Cox regression analysis with a stepwise step to screen out variables that can be used to establish a prognostic model with the coefficients weighted by the Cox model in the training cohort. Stepwise regression introduces the independent variable one by one on the condition that the independent variable is significant after the *F* test, which is commonly used to eliminate multicollinearity and select the “optimal” regression equation. In the Guangzhou training cohort, the optimal threshold was determined by using X-tile version 3.6.1, a software developed by Yale University, which allows us to divide patients into high- and low-MTBF groups. The thresholds were determined as the values that can generate the maximum chi-square values in the Mantel–Cox test (30).

To further estimate the effect of MTBF (high vs low), nodal necrosis, and nodal level, we also developed three clinical nomograms after univariate analysis. Considering the overlapping information between N stage and nodal level, we included nodal level into the backward multivariate Cox regression with other clinical factors to generate nomogram A. According to Akaike’s information criterion (AIC), sex, age, N stage, clinical stage, and LDH were selected to generate nomogram A to forecast DMFS in the training cohort. Since relevant results show that plasma EBV DNA is a prospective biomarker in NPC, we developed nomogram B by adding plasma EBV DNA. After that, we developed nomogram C based on nomogram B by integrating MTBF and nodal necrosis.

Then, calibration curves and the concordance index (C-index, proposed by Harrell) were calculated to assess and compare the prediction performance of the nomograms. To further explore the sensitivity and specificity of the prognostic model, we performed time-dependent receiver operating characteristic (ROC) analysis and calculated the area under the curve (AUC). To explore the association of nomogram C with DMFS, we calculated the total risk score for NPC patients based on nomogram C, and the patients were separated into high-risk and low-risk groups according to a cutoff value. Risk stratification was evaluated using Kaplan–Meier analysis, and the survival curves of the high-risk and low-risk groups were compared using the log-rank test. Differences in PFS between the two groups were also assessed.

The univariate and multivariate analyses of DMFS were carried out using the survival package in R (available online). With the stopping rule of AIC, the likelihood ratio test was carried out to apply backward stepwise selection. Then, the nomograms were developed by using the rms package in R (available online), including the coefficients in the multivariable Cox regression model. In this study, R version 3.5.2 and SPSS version 22.0 were adopted for statistical analyses, and a two-sided *p* value < 0.05 was adopted to indicate differences with statistical significance.

## Results

### Patient Data and Follow-Up

**FIGURE 1:**
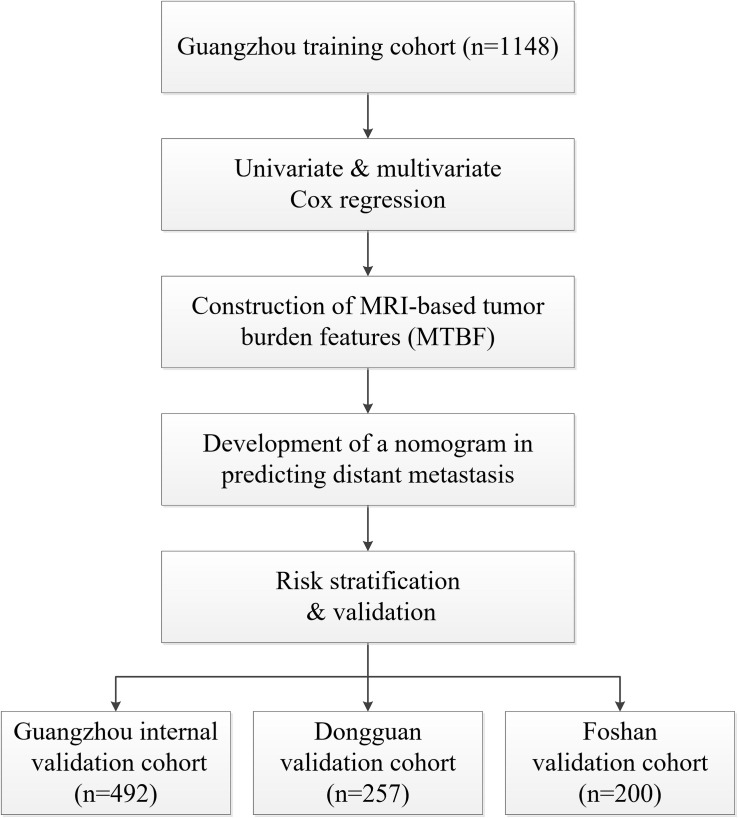
Study flow diagram. Abbreviations: MTBF, MRI-based tumor burden features.

In total, 2097 patients from three Chinese academic institutions were enrolled. The study flow diagram is shown in Figure 1. Among 1640 patients treated at Sun Yat-sen University Cancer Center, 1148 and 492 patients were randomized to a training cohort and an internal validation cohort, respectively. Additionally, we enrolled 200 and 257 patients into two independent external validation cohorts, the Foshan and Dongguan validation cohorts, respectively. The median follow-up of the combined Guangzhou cohort was 55.9 months (IQR 41.4–68.7 months), that of the Dongguan cohort was 49.2 months (IQR 37.4–60.7 months), and that of the Foshan cohort was 42.4 months (IQR 37.3–46.0 months). Up to the last follow-up, 205 (12.5%) patients in the combined Guangzhou cohort, 31 (12.1%) in the Dongguan cohort, and 19 (9.5%) in the Foshan cohort developed distant metastasis. For more information on the patients’ demographic information and baseline clinical characteristics, see Table 1.

**TABLE 1:** Baseline characteristics.

Variable	Guangzhou training set (*n* = 1148), *n* (%)	Guangzhou validation set (*n* = 492), *n* (%)	Dongguan validation set (*n* = 257), *n* (%)	Foshan validation set (*n* = 200), *n* (%)
Age (years)				
<45	541 (47.1)	250 (50.8)	104 (40.5)	82 (41.0)
≥45	607 (52.9)	242 (49.2)	153 (59.5)	118 (59.0)
Sex				
Female	280 (24.4)	112 (22.8)	62 (24.1)	42 (21.0)
Male	868 (75.6)	380 (77.2)	195 (75.9)	158 (79.0)
Tumor stage				
T1	125 (10.9)	62 (12.6)	6 (2.3)	13 (6.5)
T2	210 (18.3)	91 (10.6)	94 (36.6)	33 (16.5)
T3	620 (54.0)	250 (50.8)	120 (46.7)	97 (48.5)
T4	193 (16.8)	89 (18.1)	37 (14.4)	57 (28.5)
Node stage				
N0	178 (15.5)	96 (19.5)	27 (10.5)	42 (21.0)
N1	553 (48.2)	215 (43.7)	68 (26.5)	99 (49.5)
N2	244 (21.3)	104 (21.1)	115 (44.7)	43 (21.5)
N3	173 (15.1)	77 (15.7)	47 (18.3)	16 (8.0)
Clinical stage				
II	204 (17.8)	91 (18.5)	36 (14)	29 (14.5)
III	595 (51.8)	243 (49.4)	140 (54.5)	100 (50.0)
IV	349 (30.4)	158 (32.1)	81 (31.5)	71 (35.5)
HGB (g/L)				
<120	68 (5.9)	17 (3.5)	49 (19.1)	24 (12.0)
≥120	1080 (94.1)	475 (96.5)	208 (80.9)	176 (0.88)
LDH(U/L)				
<245	1061 (92.4)	458 (93.1)	242 (94.2)	186 (93.0)
≥245	87 (7.6)	34 (6.9)	15 (5.8)	14 (7.0)
EBVDNA level (copies/ml)				
<4000	713 (62.1)	317 (64.4)	163 (63.4)	129 (64.5)
≥4000	435 (37.9)	175 (35.6)	94 (36.6)	71 (35.5)
Nodal level				
Above the caudal edge of cricoid cartilage	979 (85.3)	420 (85.4)	212 (82.5)	180 (90.0)
Lower	169 (14.7)	72 (14.6)	45 (17.5)	20 (10.0)
Nodal necrosis				
Negative	1001 (87.2)	430 (87.4)	223 (86.8)	179 (89.5)
Positive	147 (12.8)	62 (12.6)	34 (13.2)	21 (10.5)
Tv (cm^3^)				
Median (IQR)	45.1 (30.5–73.6)	46.6 (30.6–75.4)	46.6 (32.7–83.0)	40.9 (28.7–63.5)
Lv (cm^3^)				
Median (IQR)	32.2 (5.3–76.2)	31.1 (4.0–71.2)	33.4 (12.4–69.7)	12.8 (0.3–50.3)
Ta (cm^2^)				
Median (IQR)	9.8 (7.1–14.6)	10.2 (7.3–14.8)	10.1 (7.3–15.0)	9.6 (6.7–13.6)
La (cm^2^)				
Median (IQR)	4.9 (1.2–9.2)	4.7 (1.0–8.5)	4.9 (2.5–8.3)	2.9 (0.6–7.5)
Td (cm)				
Median (IQR)	8.5 (6.6–9.9)	8.8 (7.3–9.9)	8.9 (7.3–10.5)	8.6 (7.4–10.5)
Ld (cm)				
Median (IQR)	12.1 (6.6–17.6)	12.1 (5.7–17.6)	13.0 (7.5–17.2)	9.1 (3.2–13.3)
Treatment				
CCRT	573 (49.9)	235 (47.8)	120 (46.7)	68 (34.0)
IC + CCRT	575 (50.1)	257 (52.2)	137 (53.3)	132 (66.0)

*HGB, hemoglobin; LDH, lactate dehydrogenase; EBV DNA, Epstein–Barr virus DNA; Tv, volume of the primary tumor; Lv, volume of the regional lymph nodes; Ta, maximum cross-sectional area of the primary tumor; La, maximum cross-sectional area of the regional lymph nodes; Td, vertical dimension of the primary tumor; and Ld, vertical dimension of the reginal lymph nodes.*

### Construction of the MTBF Model

In the Guangzhou training set, the first multivariate Cox regression analysis with a stepwise step showed that five of the six tumor burden variables, namely, Lv, Ta, La, Td, and Ld, were related to DMFS. In addition, a formula was generated based on the 5 tumor burden features weighted by their respective regression coefficients (Supplementary Table 1) to calculate the tumor burden score for these patients. The formula is as follows:

$$M\,T\,B\,F=-0.0043\times L_{v}+0.1121\times L_{a}+0.0587\times L_{d}+0.0528\times T_{a}+0.0917\times T_{d}$$

We generated an optimal threshold (3.2) via X-tile plots to assign the patients in the training cohort into high- and low-MTBF groups (Supplementary Figure 2). In this section, 882 (76.8%), and 266 (23.2%) patients in the training cohort, 361 (73.4%), and 131 (26.6%) patients in the Guangzhou internal validation cohort, 204 (79.4%), and 53 (20.6%) patients in the Dongguan external validation cohort, and 162 (81.0%) and 38 (19.0%) patients in the Foshan external validation cohort were divided into the high- and low-MTBF groups, respectively. The result of univariate analysis of MTBF is listed in Table 2.

**TABLE 2:** Univariate analysis of MTBF.

	DMFS			PFS		
	HR	95% CI	*p* value	HR	95% CI	*p* value
Guangzhou training cohort	4.52	3.21–6.37	<0.0001	4.22	3.22–5.54	<0.0001
Guangzhou internal validation cohort	4.77	2.98–7.65	<0.0001	3.83	2.63–5.58	<0.0001
Dongguan external validation cohort	3.13	1.52–6.46	0.0020	3.37	1.93–5.88	<0.0001
Foshan external validation cohort	5.59	2.27–13.80	0.0002	7.12	3.45–14.70	<0.0001

*MTBF, MRI-based tumor burden features; DMFS, distant metastasis-free survival; PFS, progression-free survival; HR, hazard ratio; and CI = confidence interval.*

### Development and Validation of Nomograms to Predict Survival

**FIGURE 2:**
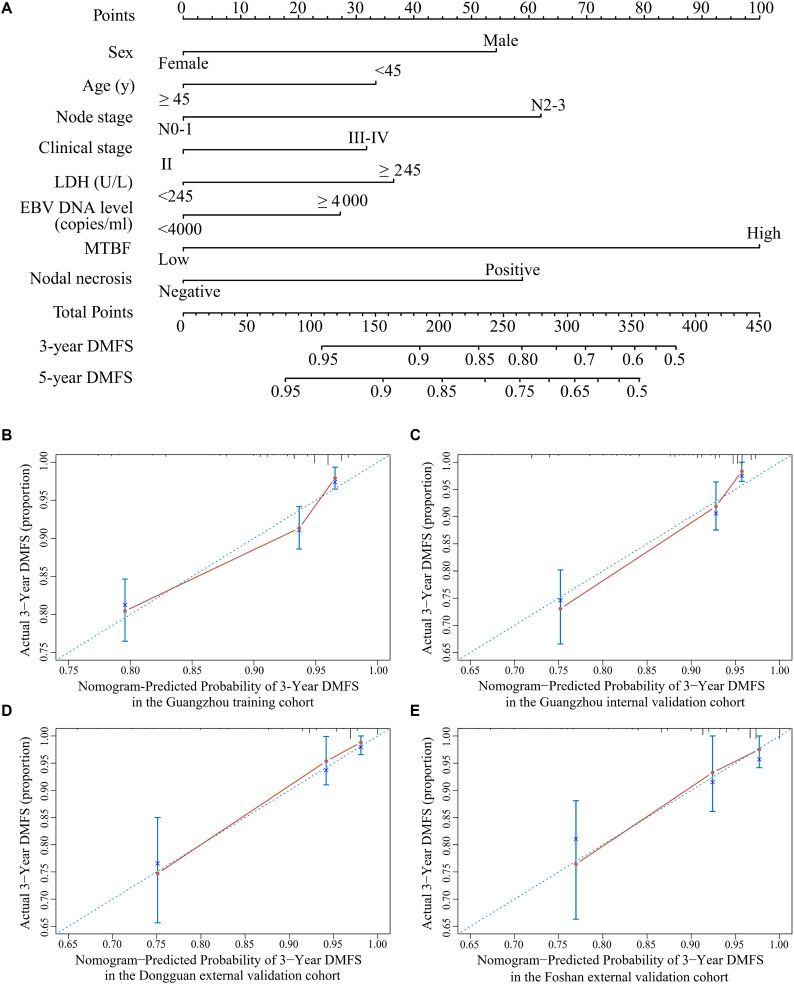
Nomogram C for predicting DMFS. **(A)** Nomogram C to predict DMFS. Calibration curves of the nomogram to predict DMFS at 3 years in **(B)** the Guangzhou training cohort, **(C)** the Guangzhou internal validation cohort, **(D)** the Dongguan external validation cohort, and **(E)** the Foshan external validation cohort. Abbreviations: LDH, lactate dehydrogenase; EBV DNA, Epstein–Barr virus DNA; MTBF, MRI-based tumor burden features; and DMFS, distant metastasis-free survival.

**FIGURE 3:**
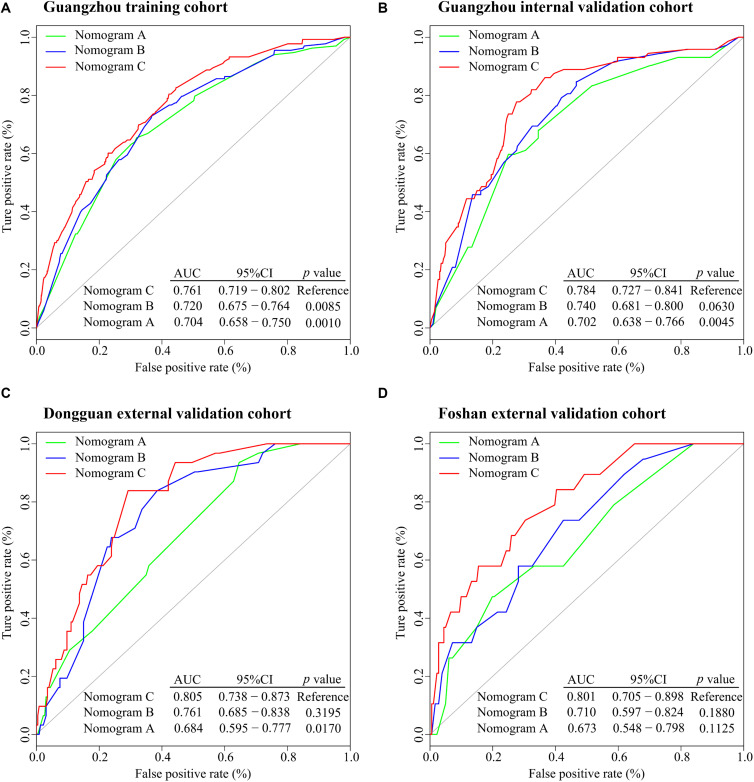
ROC analysis of nomograms **(A–C)** to compare their ability to predict DMFS. **(A)** ROC analysis in the Guangzhou training cohort. **(B)** ROC analysis in the Guangzhou internal validation cohort. **(C)** ROC analysis in the Dongguan external validation cohort. **(D)** ROC analysis in the Foshan external validation cohort. Abbreviations: ROC, receiver operating characteristic; DMFS, distant metastasis-free survival; AUC, area under the curve; and CI; confidence interval.

All variables were assessed primarily through univariate analysis for DMFS. The predictors included MTBF (high vs low), nodal necrosis (positive vs negative), nodal level (above the caudal edge of cricoid cartilage vs lower), sex, age, N stage, clinical stage, LDH, and plasma EBV DNA (Supplementary Table 2). After that, we performed multivariate analysis via a backward step using nodal level, sex, age, N stage, clinical stage, and LDH to predict the DMFS of patients in the training cohort. Sex, age, N stage, clinical stage, and LDH were ultimately selected to generate nomogram A [C-index 0.686, 95% confidence interval (CI): 0.640–0.732] (Supplementary Table 3 and Supplementary Figure 3A). Because relevant results showed that EBV DNA is a prospective prognostic biomarker of NPC, we used sex, age, N stage, clinical stage, LDH, and EBV DNA to develop nomogram B (C-index 0.702, 95% CI: 0.658–0.746; Supplementary Table 3 and Supplementary Figure 3B). Then, we used the six clinical factors, MTBF, and nodal necrosis to develop nomogram C (C-index 0.741, 95% CI: 0.702–0.780; Figure 2A). The calibration curves for 3-year DMFS also performed well in the Guangzhou internal validation set (C-index 0.738, 95% CI: 0.676–0.780), Dongguan external validation set (0.747, 0.678–0.816), and Foshan validation set (0.757, 0.657–0.857; Figures 2B–E). Then, we compared the sensitivity and specificity of the three nomograms through ROC analysis and found that nomogram C had a higher AUC value than the other two nomograms (Figure 3 and Supplementary Table 4). The ROC curve of nomogram C vs nomogram B or A reached statistical significance in the Guangzhou data set, but that of nomogram C vs B was not statistically significant in the two external validation sets.

### Nomogram C and Risk Stratification

**FIGURE 4:**
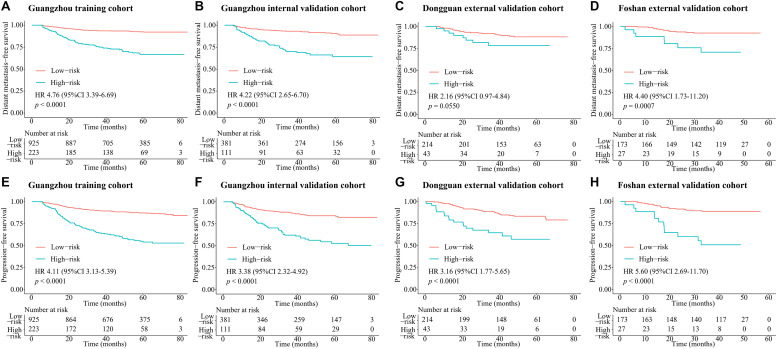
Kaplan–Meier survival curves of DMFS and PFS for risk group stratification with the MTBF. **(A)** DMFS in the Guangzhou training cohort, **(B)** DMFS in the Guangzhou internal validation cohort, **(C)** DMFS in the Dongguan external validation cohort, **(D)** DMFS in the Foshan external validation cohort, **(E)** PFS in the Guangzhou training cohort, **(F)** PFS in the Guangzhou internal validation cohort, **(G)** PFS in the Dongguan external validation cohort, and **(H)** PFS in the Foshan external validation cohort. *P* values were calculated using the unadjusted log-rank test and hazard ratios with a univariate Cox regression analysis. Abbreviations: DMFS, distant metastasis-free survival; MTBF, MRI-based tumor burden features; and HR, hazard ratio.

According to the regression coefficients of nomogram C (Supplementary Table 3), the patients’ risk scores were calculated. The patients were divided into high- and low-risk groups by the optimal cutoff value (4.1) determined by X-tile software. The 3-year DMFS rates in the high- and low-risk groups were 75.1% (95% CI: 69.5–81.0) and 93.4% (95% CI: 91.8–95.1), respectively, [hazard ratio (HR) 4.76, 95% CI: 3.39–6.69; *p* < 0.0001; Figure 4A]. Compared with low-risk patients, high-risk patients had shorter PFS (HR 4.11, 95% CI: 3.13–5.39; *p* < 0.0001; Figure 4E). In the internal validation set, 381 (77.4%), and 111 (22.6%) patients were divided into the low- and high-risk groups, respectively. The 3-year DMFS (HR 4.22, 95% CI: 2.65–6.70; *p* < 0.0001; Figure 4B) and PFS (HR 3.38, 95% CI: 2.32–4.92; *p* < 0.0001; Figure 4F) of the high- and low-risk groups were significantly different. In the Dongguan validation cohort, 214 (83.3%), and 43 (16.7%) patients were divided into the low- and high-risk groups, respectively, of which the differences in DMFS (HR 2.16, 95% CI: 0.97–4.84; *p* = 0.0550; Figure 4C) and PFS (HR 3.16, 95% CI: 1.77–5.65; *p* < 0.0001; Figure 4G) were significant. In the Foshan validation cohort, 173 (86.5%) and 27 (13.5%) of the 200 patients were assigned to the low- and high-risk groups, respectively, and significant differences were also observed. Compared with the low-risk group, the high-risk group had worse DMFS (HR 4.40, 95% CI: 1.73–11.2; *p* = 0.0007; Figure 4D) and PFS (HR 5.60, 2.69–11.7; *p* < 0.0001; Figure 4H). The numbers of events for the different risk groups in the four cohorts are listed in Supplementary Table 5.

### Nomogram C and Treatment Direction

**FIGURE 5:**
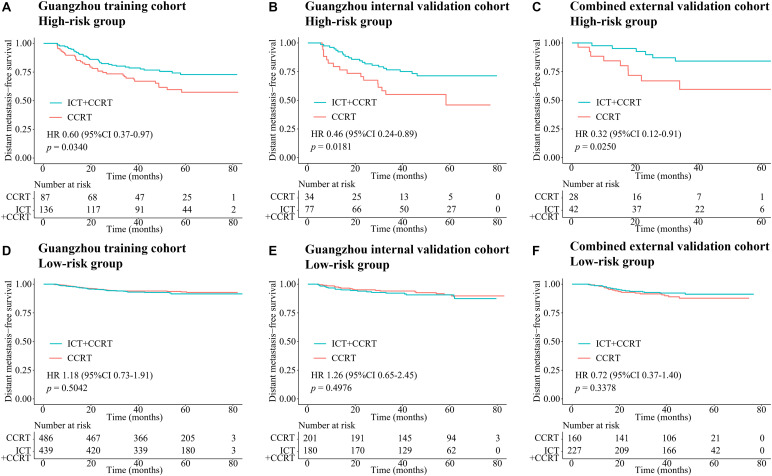
Kaplan–Meier survival curves of DMFS of CCRT alone and ICT + CCRT. **(A)** High-risk group in the Guangzhou training cohort, **(B)** high-risk group in the Guangzhou internal validation cohort, **(C)** high-risk group in the combined external validation cohort, **(D)** low-risk group in the Guangzhou training cohort, **(E)** low-risk group in the Guangzhou internal validation cohort, and **(F)** low-risk group in the combined external validation cohort. Abbreviations: DMFS, distant metastasis-free survival; CCRT, concurrent chemoradiotherapy; and ICT, induction chemotherapy.

In the Guangzhou training cohort, 486 and 439 patients in the low-risk group and 87 and 136 patients in the high-risk group received CCRT and ICT + CCRT, respectively. In the high-risk group, the DMFS of patients treated with ICT + CCRT was longer than that of those treated with CCRT alone (HR 0.60, 95% CI: 0.37–0.97, *p* = 0.0340; Figure 5A). However, the treatment effects of ICT + CCRT and CCRT were similar in the low-risk group (Figure 5D). This finding was validated in the internal and combined external validation sets (Figures 5B,C,E,F). Compared with patients not receiving ICT, among all the cohorts, treatment with ICT was also related to an improvement of PFS in patients with high-risk scores but not in those with low-risk scores (Supplementary Figure 4).

## Discussion

In this retrospective multicenter cohort study, we extracted six multidimensional tumor burden-related variables, including the volume, section area, and vertical dimension of the primary tumor and regional lymph nodes, based on which we developed the MTBF model. Compared to nomogram A (based on routine clinical features) and nomogram B (nomogram A + EBV DNA), nomogram C incorporating MTBF and nodal necrosis to nomogram B had the highest C-index (0.741, 95% CI: 0.702–0.780) and AUC value (0.761, 95% CI: 0.719–0.802). Compared with those of patients in the low-risk group stratified by nomogram C, the DMFS (HR: 4.76, 95% CI: 3.39–6.69; *p* < 0.0001), and PFS (HR: 4.11, 95% CI: 3.13–5.39; *p* < 0.0001) of patients in the high-risk group were relatively poor. Moreover, compared with patients with low risk, treatment with ICT + CCRT tended to have better effects among those with high risk.

Tumor load heterogeneity in the same T and N classification could cause much difficulty for predicting prognosis. Though the volume of the primary tumor is now a commonly acknowledged tumor burden factor for prognosis prediction (18–20), the mining of other information reflecting tumor burden remains insufficient. Our findings, which extracted tumor burden profiles such as the volume, section area, and vertical dimension of the primary tumor and regional lymph nodes based on pretreatment MRI, showed that MTBF is a promising risk factor (HR 4.52, 95% CI: 3.21–6.37; *p* < 0.0001; Supplementary Table 2). Intriguingly, Tv was removed from the construction of the MTBF model during the stepwise regression, whereas the other five tumor burden variables were retained. These results also reflect that the maximum cross-sectional area and vertical dimension could complement the effect of volume on prognosis, which offers more detailed prognostic information. In addition, compared with nomogram A (including sex, age, N stage, clinical stage, and LDH) and nomogram B (including the variables in nomogram A + EBV DNA), the proposed nomogram C (including MTBF, nodal necrosis, and the six variables in nomogram B) achieved much better performance in terms of the C-index and ROC analysis. Compared with low-risk patients, high-risk patients stratified by nomogram C had poor DMFS (HR: 4.76, 95% CI: 3.39–6.69; *p* < 0.0001) and PFS (HR: 4.11, 95% CI: 3.13–5.39; *p* < 0.0001). With the integration of tumor burden information, nodal necrosis, and other clinical characteristics, MTBF and nodal necrosis were promising supplementary factors to TNM stage and EBV DNA, yielding better performance in predicting survival. As seen in Figure 2A and the coefficients in Supplementary Table 3, MTBF played the most important role in predicting DMFS, and nodal necrosis was also a crucial variable. These results might mainly be associated with two major reasons. First, a high MTBF score and positive nodal necrosis indicate that NPC patients had unfavorable massive primary tumors or regional lymph nodes, which have a greater propensity for occult metastasis (20, 31). Second, massive tumors and positive nodal necrosis have been found to be related to the biological aggressiveness of cancer clones, inadequate blood supply, and other adverse factors, including tumor hypoxia (32, 33), which is strongly associated with radioresistance and thus relapse and metastasis.

Over 70% of NPC patients are diagnosed with an advanced stage of disease (34, 35). According to the National Comprehensive Cancer Network guidelines, locoregionally advanced NPC patients are at high risk for disease progression, for whom ICT + CCRT is recommended as level 2A evidence and CCRT alone as level 2B evidence. However, not all of these patients benefit from ICT (36). In our investigation, our proposed nomogram model C (including MTBF and nodal necrosis) performed well in predicting survival and succeeded in stratifying high- and low-risk patients who benefited from ICT + CCRT and CCRT, respectively. In the high-risk group, the DMFS of patients treated with ICT + CCRT was longer than that of patients treated with CCRT alone (HR 0.60, 95% CI: 0.37–0.97, *p* = 0.0340; Figure 5A). However, in the low-risk group, the treatment effects of ICT + CCRT and CCRT were similar. This finding might be explained by the fact that ICT not only attenuates tumor load within a brief period to ameliorate tumor hypoxia but also has a systemic cytotoxic effect to eradicate distant micrometastases (37, 38). It is well recognized that the improvement in PFS is mainly due to the reduction in distant metastases. In our study, compared with patients who did not receive ICT, patients with high-risk scores treated with ICT showed an improvement in PFS.

Magnetic resonance imaging is now extensively used for the accurate evaluation of NPC to define the TNM stage and follow-up, and the majority of NPC patients undergo MRI before treatment. Our prognostic model utilized the information obtained from pretreatment MRI without increasing the physical or financial burden. One main challenge in our study lies in the fact that three specialists in MRI are required to contour the margin of the primary tumor and regional lymph nodes, which might take time and effort. Fortunately, with the rise of artificial intelligence, deep learning has been widely applied in radiology and pathology for image processing and lesion recognition (39–42). Automatically recognizing the scope and characteristics of neoplasms will help make our proposed model a more user-friendly prognostic tool. With regard to the result of ROC analysis that the ROC curve of nomogram C vs B was not statistically significant in the two external validation sets, we think the reason lies in the different population distributions and the small sample size. Longer follow-ups and prospective multicenter clinical studies should be carried out to validate our MTBF model and nomogram. Additionally, radiomics has achieved valuable performance in many prediction tasks, including NPC (16, 43). In addition to structural tumor burden features, nodal level, and necrosis, further investigation incorporating other radiomics data will be our next research interest. In conclusion, the survival model based on MTBF, nodal necrosis, and clinical factors is a promising prognostic tool for NPC and is helpful for identifying patients who might benefit from ICT.

## Data Availability Statement

The datasets generated for this study are available on request to the corresponding author.

## Ethics Statement

This study was approved by the Ethics Committee of the Chinese Clinical Trial Registry (ChiECRCT20190127). Patients’ written informed consent was not required because no direct interaction with patients was performed and no personal identification was applied in this study. In addition, this research was conducted in compliance with the Declaration of Helsinki.

## Author Contributions

XL, CFL, and XiC designed this study. XiC, MQ, GL, JL, MG, ZC, JM, SL, and WX collected data. LK, XuC, KL, YX, and XG rechecked data. XiC, XuC, BJ, and WL performed analysis and wrote the manuscript. CXL, CFL, and XL helped to revise the manuscript. All authors approved the final version of the manuscript.

## Conflict of Interest

The authors declare that the research was conducted in the absence of any commercial or financial relationships that could be construed as a potential conflict of interest.
