# Supplementary material for: Prognostic and Treatment Guiding Significance of MRI-Based Tumor Burden Features and Nodal Necrosis in Nasopharyngeal Carcinoma
Source: Front Oncol. 2020 Sep 11;10:537318. doi: 10.3389/fonc.2020.537318 (PMC7518313; doi:10.3389/fonc.2020.537318)
Supplement: Supplementary file 1 [file Data_Sheet_1.docx]

Supplementary Material

# Supplementary Data

**Inclusion criteria**

The inclusion criteria were as follows:

1. patients with histologically confirmed non-metastatic NPC;
2. patients who received radiotherapy;
3. patients who underwent concurrent chemoradiotherapy (CCRT) with or without IC;
4. patients who underwent pretreatment head and neck MRI; and
5. patients aged between 18-70 years regardless of sex.

**Exclusion criteria**

The exclusion criteria were as follows:

1. patients with a history of other malignancies;
2. presence of a primary distant metastasis;
3. patients with crucial organ dysfunction; and
4. patients without an effective and accurate follow-up.

**MRI execution**

Unenhanced and enhanced head and neck MR scans were accomplished in the [supine](javascript:;) [position](javascript:;). Covering a region from the frontal sinuses to the lower edge of the sternal collarbone, MR scans were performed on a 1.5T or 3.0T imaging system (GE Discovery MR 750 3.0T, USA; Siemens MAGNETON Trio A Tim, 3.0T, German; Signa HDx Echospeed 1.5T, USA). Before injecting Gd-DTPA via the superficial vein, T1-weighted imaging (T1WI) was performed in the axial, coronal and sagittal planes, and T2-weighted imaging (T2WI) was performed in the axial plane. After Gd-DTPA injection with a dose of 0.3-0.4 mmol per kg body weight, T1WI in the axial, coronal and sagittal planes and T2WI in the axial plane were immediately executed.

**Calculation of tumour burden features (*Tv*, *Lv*, *Ta*, *La*, *Td*, and *Ld*)**

Based on enhanced T1WI, three specialists in MRI contoured the full extent of the primary tumour and regional lymph nodes by utilizing Medical Imaging Interaction Toolkit (MITK) software (version MITK-2016.11.0). Disagreement was solved by a consensus. Quantification of the vertical dimension, maximum cross-sectional area, and volume of the primary tumour and regional lymph nodes were all derived from the contoured profiles.

1. The vertical dimension (*d*) was calculated by adding the image slice thickness (Thickness) to the [interlayer](javascript:;) [distance](javascript:;) (*D*[*_interlayer_*](javascript:;)) and then multiplying by the layer number (*Num_layer_*).

$$\boldsymbol{d=Nu}\boldsymbol{m}_{\boldsymbol{layer}}\boldsymbol{*Thickness*}\boldsymbol{D}_{\boldsymbol{interlayer}}$$

2. The maximum cross-sectional area (*a*) was defined as the largest one of every cross-sectional area (*a_i_*), which was obtained by calculating the product of the voxel number (*M_i_*) and the length (*Length_voxel_*) and width (*Width_voxel_*) of a single voxel.

$$\boldsymbol{a}_{\boldsymbol{i}}\boldsymbol{=}\boldsymbol{M}_{\boldsymbol{i}}\boldsymbol{*}\boldsymbol{Length}_{\boldsymbol{voxel}}\boldsymbol{*}\boldsymbol{Width}_{\boldsymbol{voxel}}$$

$$\boldsymbol{a=Maximum(}\boldsymbol{a}_{\boldsymbol{i}}\boldsymbol{)}$$

3. The volume (*v*) was calculated as the sum of the slice volumes and interlayer volumes.

Specifically, the slice volume (*v_s_*) was the product of the voxel number, single voxel area and slice thickness.

$$\boldsymbol{v}_{\boldsymbol{s}}\boldsymbol{=}\sum_{\boldsymbol{i}}^{\boldsymbol{Num}_{\boldsymbol{layer}}} \boldsymbol{M}_{\boldsymbol{i}}\boldsymbol{*}\boldsymbol{Length}_{\boldsymbol{voxel}}\boldsymbol{*}\boldsymbol{Width}_{\boldsymbol{voxel}}\boldsymbol{*Thickness}$$

The interlayer volume (*v_interlayer_*) was the sum of the average area of two adjacent slices multiplied by the interlayer distance.

$$\boldsymbol{v}_{\boldsymbol{interlayer}}\boldsymbol{=}\sum_{\boldsymbol{i}}^{\boldsymbol{Num}_{\boldsymbol{layer}}} \left( \frac{\boldsymbol{M}_{\boldsymbol{i}}\boldsymbol{+}\boldsymbol{M}_{\boldsymbol{i+1}}}{\boldsymbol{2}} \right)\boldsymbol{*}\boldsymbol{Length}_{\boldsymbol{voxel}}\boldsymbol{*}\boldsymbol{Width}_{\boldsymbol{voxel}}\boldsymbol{*}\boldsymbol{D}_{\boldsymbol{interlayer}}$$

Therein, the volume (v) was calculated as the sum of the slice volumes and interlayer volumes.

$$\boldsymbol{v=}\boldsymbol{v}_{\boldsymbol{s}}\boldsymbol{+}\boldsymbol{v}_{\boldsymbol{interlayer}}\boldsymbol{=}\sum_{\boldsymbol{i}}^{\boldsymbol{Num}_{\boldsymbol{layer}}} \boldsymbol{M}_{\boldsymbol{i}}\boldsymbol{*}\boldsymbol{Length}_{\boldsymbol{voxel}}\boldsymbol{*}\boldsymbol{Width}_{\boldsymbol{voxel}}\boldsymbol{*Thickness+}\sum_{\boldsymbol{i}}^{\boldsymbol{Num}_{\boldsymbol{layer}}} \left( \frac{\boldsymbol{M}_{\boldsymbol{i}}\boldsymbol{+}\boldsymbol{M}_{\boldsymbol{i+1}}}{\boldsymbol{2}} \right)\boldsymbol{*}\boldsymbol{Length}_{\boldsymbol{voxel}}\boldsymbol{*}\boldsymbol{Width}_{\boldsymbol{voxel}}\boldsymbol{*}\boldsymbol{D}_{\boldsymbol{interlayer}}$$

# Supplementary Figures and Tables

## Supplementary Figures


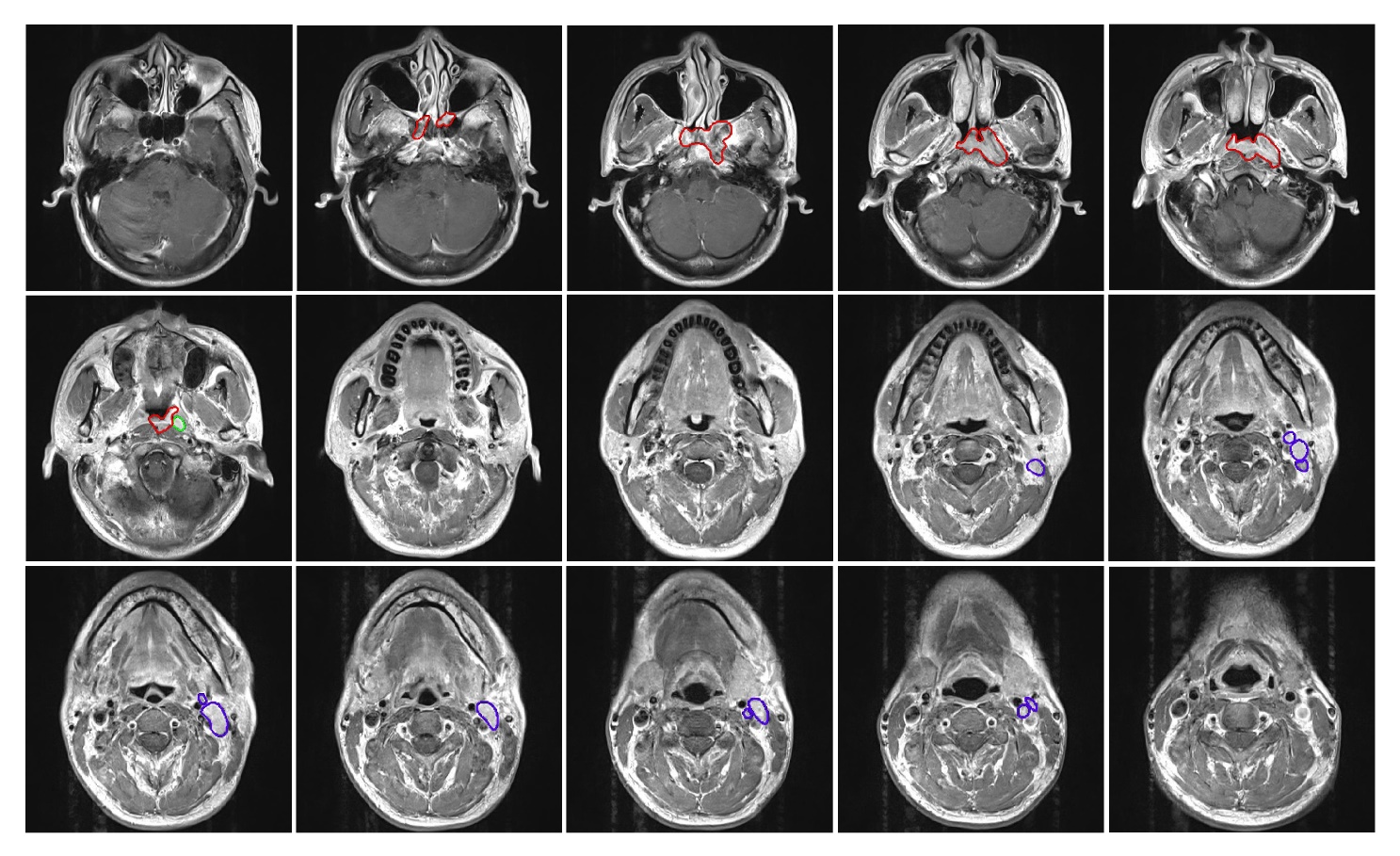


**Supplementary Figure 1 An example of the full extent of the primary tumor and regional lymph nodes.**

The full extent of the primary tumor and regional lymph nodes was contoured by three specialists in MRI utilizing the MITK software**.** MITK = Medical Imaging Interaction Toolkit.


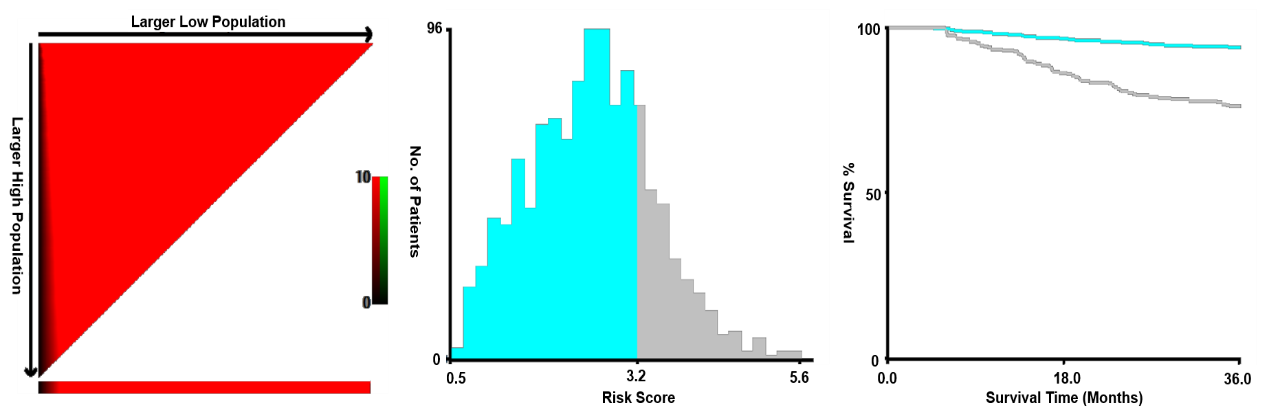


**Supplementary Figure 2 Generation of the optimum cutoff value for the MTBF.**

In the Guangzhou training cohort, we used X-tile software to select the optimal MTBF cutoff value. The colors shown in the plot represent the strength of the association at each division. Red indicates an inverse association between the risk score and DMFS, whereas green represents a direct association. Risk score 3.2 was the optimal cutoff value for the MTBF, which separated the survival curves significantly. MTBF = MRI-based tumor burden features. DMFS = distant metastasis-free survival.


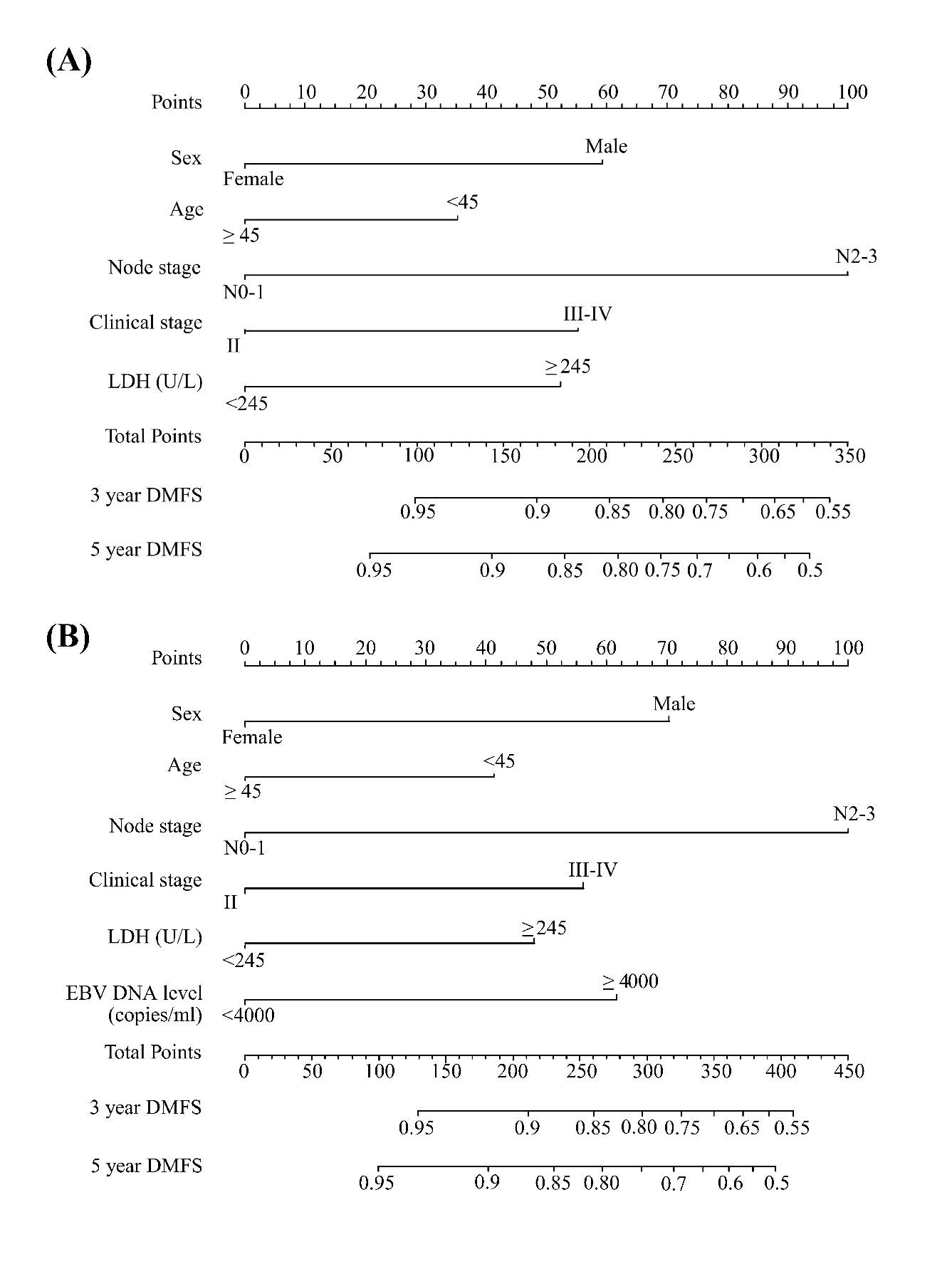


**Supplementary Figure 3 Nomogram A and B for DMFS.**

**(A)** Nomogram A for DMFS recruiting gender, age, node stage, clinical stage, and LDH, and **(B)** nomogram B for DMFS recruiting gender, age, node stage, clinical stage, LDH, and EBV DNA. LDH = lactate dehydrogenase. EBV DNA = Epstein-Barr virus DNA. DMFS = distant metastasis-free survival.


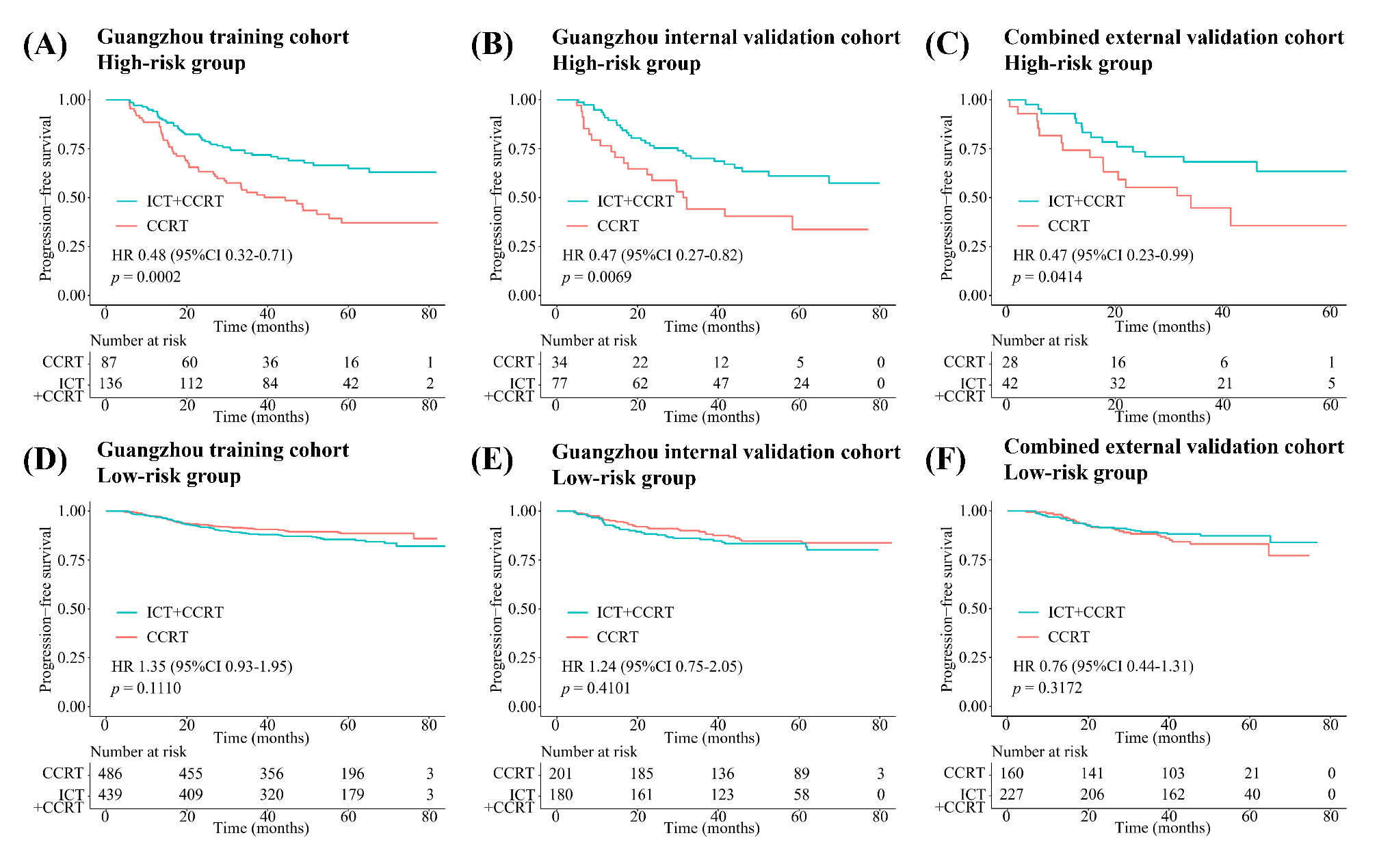


**Supplementary Figure 4 Kaplan-Meier survival curves of PFS between CCRT alone and ICT+CCRT**.

**(A)** High-risk group in the Guangzhou training cohort, **(B)** high-risk group in the Guangzhou internal validation cohort, **(C)** high-risk group in the combined external validation cohort, **(D)** low-risk group in the Guangzhou training cohort, **(E)** low-risk group in the Guangzhou internal validation cohort, and **(F)** low-risk group in the combined external validation cohort. PFS = progression-free survival. CCRT = concurrent chemoradiotherapy. ICT = induction chemotherapy.

## Supplementary Tables

**Supplementary Table 1: Coefficients for the MTBF prediction algorithm.**

|  | coef | se (coef) | z | ***p* value** |
| --- | --- | --- | --- | --- |
| Lv | -0.0043 | 0.0027 | -1.57 | 0.1161 |
| La | 0.1121 | 0.0331 | 3.39 | 0.0007 |
| Ld | 0.0587 | 0.0190 | 3.09 | 0.0007 |
| Ta | 0.0528 | 0.0144 | 3.67 | 0.0002 |
| Td | 0.0917 | 0.0389 | 2.36 | 0.0185 |

Abbreviation: MTBF = MRI-based tumor burden features. Lv = volume of the reginal lymph nodes. Ta = maximum cross-sectional area of the primary tumor. La = maximum cross-sectional area of the reginal lymph nodes. Td = vertical dimension of the primary tumor. Ld = vertical dimension of the reginal lymph nodes.

**Supplementary Table 2: Univariate analysis of prognostic factors of DMFS in the Guangzhou training cohort.**

| **Variables** | **HR** | **95%CI** | ***p* value** |
| --- | --- | --- | --- |
| Sex (male vs. female) | 1.75 | 1.10-2.80 | **0.0181** |
| Age (≥ 45 vs. < 45) | 0.751 | 0.53-1.06 | **0.0988** |
| N stage (N2-3 vs. N0-1) | 3.25 | 2.29-4.63 | **<0·0001** |
| Clinical stage (Ⅱ vs. Ⅲ-Ⅳ) | 3.12 | 1.59-6.14 | **0.0010** |
| LDH (≥ 245 vs. < 245 U/L) | 2.21 | 1.36-3.59 | **0.0014** |
| EBV DNA level (≥4000 vs. <4000 (copies/ml) | 2.46 | 1.74-3.47 | **<0·0001** |
| Nodal necrosis (positive vs negative) | 2.43 | 1.66-3.55 | **<0·0001** |
| Nodal level (above the caudal edge of cricoid cartilage vs lower) | 2.70 | 1.84-3.96 | **<0·0001** |
| MTBF (high vs. low) | 4.52 | 3.21-6.37 | **<0·0001** |

Abbreviations: HR = hazard ratio; CI = confidence interval. LDH = lactate dehydrogenase. EBV DNA = Epstein-Barr virus DNA. MTBF = MRI-based tumor burden features.

**Supplementary Table 3: Coefficients for the nomogram A, B, C.**

|  | coef | se (coef) | z | ***p* value** |
| --- | --- | --- | --- | --- |
| **Nomogram A** | | | | |
| Sex (male vs. female) | 0.6051 | 0.2392 | 2.53 | 0.0114 |
| Age (≥ 45 vs. < 45) | -0.3597 | 0.1749 | -2.06 | 0.0397 |
| N stage (N2-3 vs. N0-1) | 1.0202 | 0.1934 | 5.28 | <0.0001 |
| Clinical stage (Ⅱ vs. Ⅲ-Ⅳ) | 0.5637 | 0.3690 | 1.53 | 0.1266 |
| LDH (≥ 245 vs. < 245 U/L) | 0.5337 | 0.2507 | 2.13 | 0.0332 |
| **Nomogram B** | | | | |
| Sex (male vs. female) | 0.6175 | 0.2392 | 2.58 | 0.098 |
| Age (≥ 45 vs. < 45) | -0.3627 | 0.1750 | -2.07 | 0.0381 |
| N stage (N2-3 vs. N0-1) | 0.8774 | 0.1996 | 4.40 | <0.0001 |
| Clinical stage (Ⅱ vs. Ⅲ-Ⅳ) | 0.4926 | 0.3700 | 1.33 | 0.1831 |
| LDH (≥ 245 vs. < 245 U/L) | 0.4212 | 0.2533 | 1.66 | 0.0964 |
| EBV DNA level (≥4000 vs. <4000 (copies/ml) | 0.5408 | 0.1875 | 2.88 | 0.0039 |
| **Nomogram C** | | | | |
| Sex (male vs. female) | 0.5113 | 0.2407 | 2.12 | 0.0336 |
| Age (≥ 45 vs. < 45) | -0.3146 | 0.1750 | -1.80 | 0.0722 |
| N stage (N2-3 vs. N0-1) | 0.5843 | 0.2063 | 2.83 | 0.0046 |
| Clinical stage (Ⅱ vs. Ⅲ-Ⅳ) | 0.2989 | 0.3751 | 0.80 | 0.4255 |
| LDH (≥ 245 vs. < 245 U/L) | 0.3437 | 0.2528 | 1.36 | 0.1740 |
| EBV DNA level (≥4000 vs. <4000 (copies/ml) | 0.2564 | 0.1949 | 1.32 | 0.1884 |
| MTBF (high vs. low) | 0.9416 | 0.2034 | 4.63 | <0.0001 |
| Nodal necrosis (positive vs negative) | 0.5543 | 0.2029 | 2.73 | 0.0063 |

Abbreviations: LDH = lactate dehydrogenase. EBV DNA = Epstein-Barr virus DNA. MTBF = MRI-based tumor burden features.

|  | Guangzhou training cohort | | | Guangzhou internal validation cohort | | | Dongguan external validation cohort | | | Foshan external validation cohort | | |
| --- | --- | --- | --- | --- | --- | --- | --- | --- | --- | --- | --- | --- |
|  | AUC | 95%CI | *p* value | AUC | 95%CI | *p* value | AUC | 95%CI | *p* value | AUC | 95%CI | *p* value |
| Nomogram A | 0.704 | 0.658-0.750 | 0.0010 | 0.702 | 0.638-0.766 | 0.0045 | 0.684 | 0.595-0.777 | 0.0170 | 0.673 | 0.548-0.798 | 0.1125 |
| Nomogram B | 0.720 | 0.675-0.764 | 0.0085 | 0.740 | 0.681-0.800 | 0.0630 | 0.761 | 0.685-0.838 | 0.3195 | 0.710 | 0.597-0.824 | 0.1880 |
| Nomogram C | 0.761 | 0.719-0.802 | Reference | 0.784 | 0.727-0.841 | Reference | 0.805 | 0.738-0.837 | Reference | 0.801 | 0.705-0.898 | Reference |

**Supplementary Table 4: AUC of the nomogram A, B, C.**

Abbreviations: AUC = area under the curve. CI = confidence interval.

| Number of events | Guangzhou training cohort  (n=1148) | |  | Guangzhou internal validation cohort  (n=492) | |  | Dongguan external validation cohort  (n=257) | |  | Foshan external validation cohort  (n=200) | |
| --- | --- | --- | --- | --- | --- | --- | --- | --- | --- | --- | --- |
|  | Low-risk  (n=925) | High-risk  (n=223) |  | Low-risk  (n=381) | High-risk  （n=111） |  | Low-risk  (n=214) | High-risk  （n=43） |  | Low-risk  (n=173) | High-risk  （n=27） |
| Distant metastasis | 66 | 67 |  | 35 | 37 |  | 23 | 8 |  | 12 | 7 |
| Progression | 115 | 95 |  | 60 | 50 |  | 35 | 17 |  | 18 | 12 |
| Death | 32 | 35 |  | 17 | 21 |  | 24 | 15 |  | 20 | 12 |

**Supplementary Table 5: Number of events for the high-risk and low-risk groups.**
